# Mixed hepatoblastoma and teratoma of the liver in a 3-year-old child: a unique combination and clinical challenge

**DOI:** 10.1186/1746-1596-4-37

**Published:** 2009-11-12

**Authors:** Alexander Moll, Alexander Krenauer, Uta Bierbach, Holger Till, Wolfgang Hirsch, Ivo Leuschner, Nicole Schmitz, Christian Wittekind, Thomas Aigner

**Affiliations:** 1 Institute of Pathology, University of Leipzig, Liebigstrasse 26, 04103 Leipzig, Germany; 2 Department of Pediatrics, University of Leipzig, Liebigstrasse 20a, 04103 Leipzig, Germany; 3 Department of Pediatric Surgery, University of Leipzig, Liebigstrasse 20a, 04103 Leipzig, Germany; 4 Department of Radiology, University of Leipzig, Liebigstrasse 20, 04103 Leipzig, Germany; 5 Institute of Pathology, UKSH-Campus Kiel, Michaelisstrasse 11, 24105 Kiel, Germany

## Abstract

Primary liver tumors in children are rare with malignant hepatoblastoma being the most common neoplasm. In this report, we describe the diagnosis and clinical management of a large liver tumor in a 3-year-old child that displayed the features of both, conventional hepatoblastoma and malignant teratoma. Pathological assessment on a pre-operative bioptical specimen showed an immature teratoid tumor with no area of hepatoblastic differentiation present. Histological and immunhistological examination of the resected tumor specimen additionally showed tumor areas of very different differentiation pattern intermixed with each other, namely areas of hepatoblastoma-typical and neuroblastoma-like morphology as well as areas of rhadomyosarcomatous differentiation.

After chemotherapy the tumor size increased and an extended right hemihepatectomy was performed. Post-operatively, the general condition of the child improved and adjuvant chemotherapy was started two weeks later. 36 months after initial diagnosis the patient is healthy, in good general condition, and without any sign of residual tumor disease.

Overall, we describe the diagnosis and clinical management of a large liver tumor in a 3-year-old child that displayed the features of both, conventional hepatoblastoma and malignant teratoma and was designated as mixed hepatoblastoma and teratoma. Though mesenchymal tumor portions can occur within hepatoblastomas, most commonly osteoid or chondroid, our case is different as it presents a large spectrum of mesenchymal and epithelial differentiation pattern in most of the lesion.

## Background

Primary liver tumors in children are rare. The liver is embryologically derived from both meso- and endodermal tissues and, thus, can develop a wide variety of both benign and malignant neoplasms. Hepatoblastoma is the most common malignant hepatic neoplasm of childhood, representing about 75% of the primary liver tumors in children in Western countries. Hepatic (benign or malignant) teratoma is an extremely rare tumor with only single cases reported world-wide [1-4]. In this report, we describe the diagnosis and clinical management of a mixed hepatoblastoma and teratoma of the liver in a 3-year-old boy.

## Clinical History and Histological Findings

**Figure 1:**
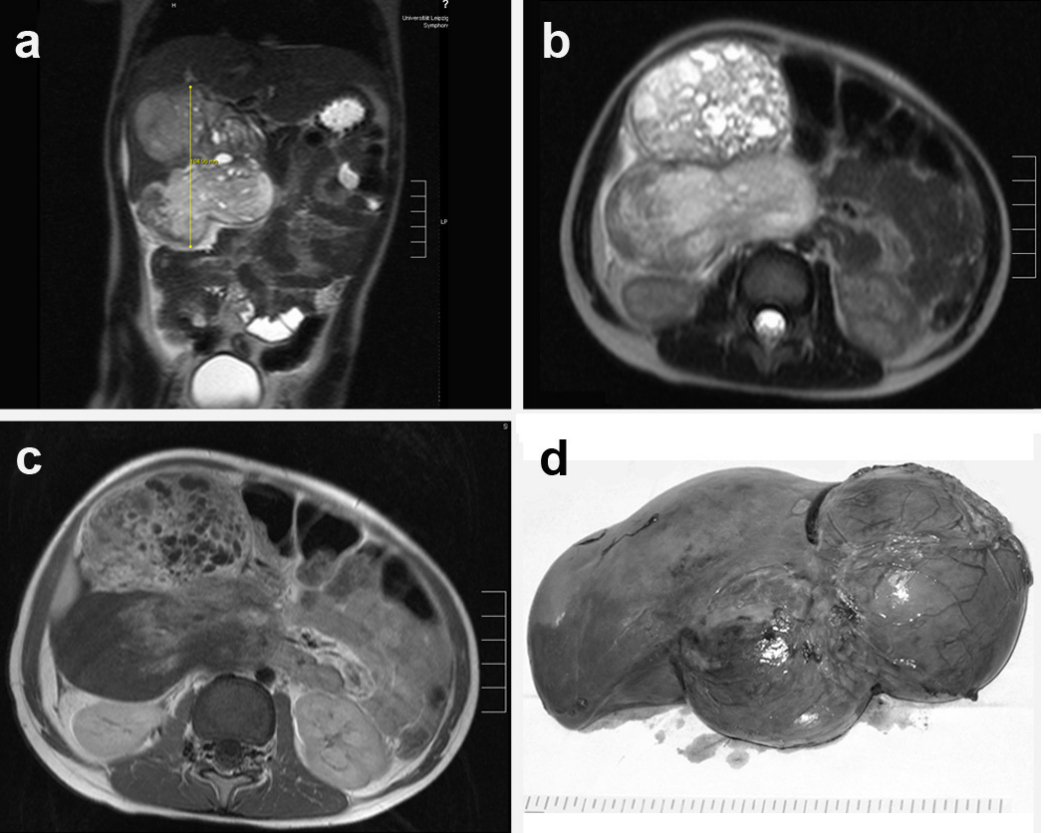
**MRI shows a large tumor in the right upper abdomen originating from the right liver lobe**. The tumor has two main parts and seems to contain different parts of tissue. The coronal view showed that the tumor is mainly situated outside the liver (a: T2 sequence). The transversal view reveals that the ventral tumor part has a cystic appearance, whereas the dorsal part appeared more solid (b: T2 sequence). The T1 sequence after contrast application (c) showed an inhomogeneous contrast enhancement. The ventral part of the tumor is enhancing a lot, the dorsal solid part does not enhance in the lateral regions. **d: **Macroscopic appearance of the resected tumor specimen.

A critically ill 3-year-old boy presented with a hard mass beneath the right costal margin. Magnetic resonance tomography showed a large, inhomogeneous mass measuring 10 × 9.5 × 7.3 cm with an intra- and extra-hepatic portion (fig. 1a-c) and a partial compression of the vena cava inferior, but no metastatic disease. Laboratory tests revealed an increase of liver transaminases and a strong increase of alpha-fetoprotein (AFP) and neuron-specific enolase.

Pathological assessment on a bioptical specimen showed an immature teratoid tumor with no area of hepatoblastic differentiation present. After a 5-day PEI chemotherapy following the MAKEI 96 protocol of the German Society for Pediatric Oncology and Hematology (GPOH) [5] AFP decreased, but the tumor size increased to 14 × 14 × 20 cm. An extended right hemihepatectomy was performed. The gross surgical specimen measured 23.5 × 16.8 × 11 cm (1915 g) with a lesion size of 16 × 14 × 11 cm (fig. 1d) and a minimal tumor-free margin of 0.3 cm. The tumor was encapsulated and macroscopically showed multiple cysts with a diameter of up to 1.5 cm, about 40% of the tumor was necrotic.

The patient recovered well and showed no signs of further tumor disease 36 months postoperatively.

**Figure 2:**
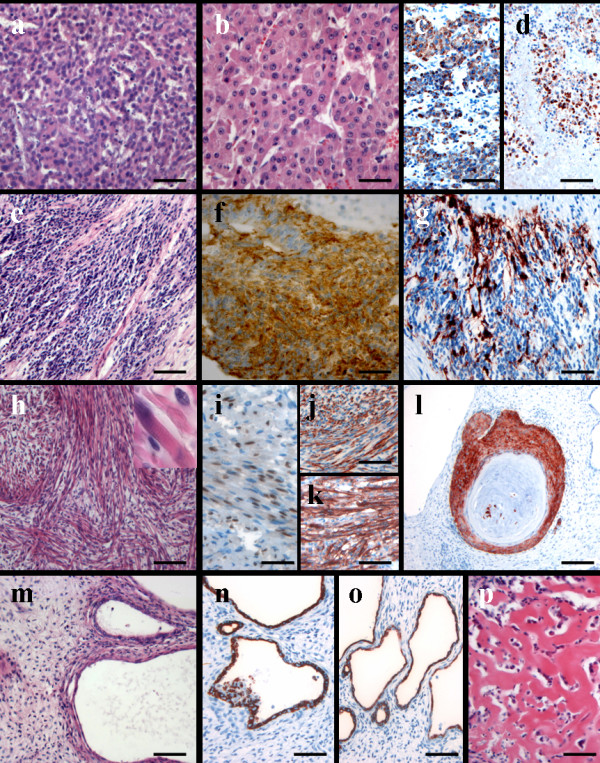
**Conventional HE-staining demonstrated areas of embryonic (a) and fetal (b) hepatoblastic cells, which were positive for HepPar-1 (c)**. Of note, the tumor cells in the necrotic tumor portions were also focally still HepPar-1 positive (d). Other tumor areas showed tumor cells embedded into a fibrillar matrix (e) and revealed a strong positivity for synaptophysin (f) as well as partly S-100 protein (g). In other areas a rhabdomyosarcomatous tumor cell differentiation (h; insert showing focally visible cross-striation) with expression of myogenin (i), desmin (j) as well as sarcomer actin (k) was visible. Cytokeratin 5/6 expression was restricted to small foci of squameous differentiation (l). Areas of cystic epithelial differentiation (m) showed positivity for cytokeratins 7 (n) and 19 (o). Focally, osteoid formation was observed (p). (a,b,e,h,m,p: hematoxylin eosin; c,d,g,h,i-l,n,o: immunostainings with antibodies as indicated). (magnification bars a,b,i: 50 μm; c-h,j-p: 100 μm; l: 300 μm)

Histological examination showed tumor areas of very different differentiation pattern intermixed with each other (i. e. not representing a collision tumor). Roughly, a fifth of the examined tumor displayed a hepatoblastoma-typical morphology partly resembling embryonic and fetal hepatoblastoma (fig. 2a, b). About 15% demonstrated a neuroblastoma-like morphology (fig. 2e) and 5-10% spindle-shaped areas with cross-striation being visible in single cells (fig. 2h). The remaining tumor showed necrotic tissue or grossly fibro-myxoid stroma-like areas as well as multifocally mostly cystic epithelial structures (fig. 2m). Rarely, squameous differentiation or osteoid formation (fig. 2o, p) was seen.

Immunohistochemical analysis (antibodies are listed in table 1; results in table 2) revealed expression of HepPar-1 selectively in the hepatoblastic cells (fig. 2c) including a significant amount of the cells within the necrotic tumor areas (fig. 2d) suggesting that mostly the hepatoblastic tumor portions were showing regression after chemotherapy. No expression of AFP was found in any tumor portion. The spindle-shaped cells presenting focally cross-striation (fig. 2h: insert) expressed strongly desmin (fig. 2j), sarcomer actin (fig. 2k) and partly myogenin (fig. 2i) in line with the rhabdomyosarcomatous phenotype of the cells. The gland-like structures expressed epithelial cytokeratins (KL-1), particularly cytokeratins 7 (fig. 2n) and 19 (fig. 2o) similar to bile duct epithelia. CK5/6 was specifically positive in the areas of squameous differentiation (fig. 2l). The small cell tumor component embedded in a neurofibril-like material was positive for synaptophysin (fig. 2f), neuron-specific enolase (NSE), S-100 protein (fig. 2g), and CD56, which confirmed its neuroblastic differentiation. CD-99 as well as TTF-1 (thyroid transcription factor-1) was negative. The proliferation rate was 5 to 10% except for in the spindle-cell areas, which showed an increased proliferation rate (up to 30%).

FISH analysis demonstrated no amplification of the MYCN-oncogene, which would have been an indicator of a poor prognosis.

Post-operatively, the general condition of the child improved and the first of four 10-day IPA chemotherapy cycles following the HB 99 protocol of the GPOH was started two weeks later. 36 months after initial diagnosis the patient is healthy, in good general condition, and without any sign of residual tumor disease.

## Discussion

Hepatoblastoma is a rare, but the most common hepatic malignancy of childhood with a peak incidence from 6 months to 3 years. The etiology of hepatoblastoma is unknown, but it has been associated with Wiedemann-Beckwith syndrome, familial adenomatosis polyposis coli, prematurity and low birth weight. Hepatoblastomas are well-defined, solid, mostly intrahepatical lesions. Lymphatic and hematogenic metastases occur in advanced disease stages with the latter mostly involving the lungs. AFP levels are nearly always elevated in hepatoblastomas and are correlated in most cases with the stage of the disease [6]; also the rate of decline of AFP during treatment is of prognostic value. The decrease of AFP during chemotherapy, despite an increase in tumor size in our case, is at first sight unusual, but most likely reflects a rather high chemosensitivity of the hepatoblastoma tumor portions. In the teratoma, areas kept proliferating and were hardly affected. Correspondingly, the cells in the areas of tumor necrosis were at least in part still positive for HepPar-1.

Teratomas are rare neoplasms (incidence 0.7/100.000 children/year) with tissue derivatives of all three germ layers. Teratomas mostly occur in the ovaries, the sacrococcygeal region, the testes, and the central nerval system and only rarely in other locations with less than 5% occurring in the abdomen [7]. Teratomas are thought to have been present since birth, or even before birth, and are therefore considered as congenital tumors. In the liver, only single cases of (benign or malignant) teratomas have been described [1-4].

Our case represents a nearly unique combination of both tumor entities, hepatoblastoma and malignant teratoma in a young boy. Though mesenchymal tumor portions can occur within hepatoblastomas, most commonly osteoid or chondroid [8], our case is different as it presents a large spectrum of mesenchymal and epithelial differentiation pattern in most of the lesion. Obviously, the dispute whether to call such a lesion mixed hepatoblastoma and teratoma or teratoid hepatoblastoma [8] might be mostly semantic. In our case, in which most of the lesion represented teratoma, however, we clearly prefer to talk about a mixed or combined neoplasm, namely mixed hepatoblastoma and teratoma.

The treatment of the presented mixed hepatoblastoma and teratoma of the liver was based on a combined systemic chemotherapy and surgery. Therapies of such a mixed tumor tissue have not been described yet, but children with hepatoblastomas have nowadays, due to the new therapy modalities, a rather good prognosis with a 5-year survival rate of over 70% [9] with curative surgery being the primary treatment of all pediatric liver tumors especially in the absence of metastatic disease. Systemic chemotherapy is beneficial as metastases are detectable at diagnosis in about 20% of all patients and most children suffer tumor recurrence after surgery alone.

In summary, we report on the diagnosis and clinical management of to our knowledge the second case of a mixed hepatoblastoma and teratoma in a young boy [10]. Clearly, this represents a rare facet of embryonic tumors within the liver. This case as well as similar cases with a very peculiar tumor biology [11] documents the importance of adequate sampling of tumor material in all cases of heterogenous tumor differentiation in order not to miss minor, but relevant tumor portions. Management, as far as it can be estimated from a single case, appears to be along the guidelines valid for hepatoblastoma alone with, however, the caveat that the (applied) chemotherapy was only effective in the hepatoblastoma areas and not the teratoma portion of the tumor.

## Competing interests

The authors declare that they have no competing interests.

## Authors' contributions

AM collected data and was the main author of the manuscript. AK helped with evaluation of the data. UB was the treating pediatric oncologist, HT the operating surgeon, WH the responsible radiologist, IL the consiliary pathologist, NS helped writing the paper and CW and TA were the local pathologists making the primary diagnosis. TA was the senior supervisor of the work performed. All authors critically reviewed the manuscript.
